# Single-Cell Transcriptomic Analysis Identifies an OLFM4-Associated Gastric Cancer Cell State with Palmitoylation-Related Signatures and Altered Metabolic Activities

**DOI:** 10.3390/biom16060880

**Published:** 2026-06-15

**Authors:** Gong Chen, Weiping Wei, Dan Li, Shanshan Han, Michael Schäfer, Xiaoyan Huang

**Affiliations:** 1 Department of General, Visceral and Transplantation Surgery, University of Heidelberg, 69120 Heidelberg, Germany; gong.chen@stud.uni-heidelberg.de (G.C.); dan.li@stud.uni-heidelberg.de (D.L.); shanshan.han@stud.uni-heidelberg.de (S.H.); michael.schaefer@exchi.uniheidelberg.de (M.S.); 2 First Department of Medicine, Medical Faculty Mannheim, University Medical Centre Mannheim (UMM), Heidelberg University, 68167 Mannheim, Germany; weiping.wei@stud.uni-heidelberg.de

**Keywords:** gastric cancer, single-cell RNA sequencing, tumor heterogeneity, OLFM4, palmitoylation-related signatures, metabolic activities

## Abstract

Gastric adenocarcinoma (STAD) exhibits extensive intratumoral heterogeneity that contributes to tumor progression and therapeutic resistance. In this study, we integrated single-cell RNA sequencing and bulk transcriptomic analyses to characterize malignant epithelial subtypes in STAD. Among seven identified tumor subtypes, the OLFM4-associated C3 subtype exhibited enriched palmitoylation-related signatures and altered metabolic activities, particularly glycolysis-related pathways. Functional enrichment analyses further supported the enrichment of multiple energy metabolism pathways. To evaluate the association between OLFM4 and metabolic regulation, recombinant OLFM4 treatment and siRNA-mediated OLFM4 knockdown were performed in gastric cancer cell lines. OLFM4 upregulation increased the expression of ZDHHC2 and GLUT1, accompanied by enhanced glucose uptake and elevated ATP production, whereas OLFM4 silencing reduced ZDHHC2 and GLUT1 expression. In addition, a prognostic risk model derived from C3 subtype-associated genes (MUC16, RALA, and PCBD1) effectively stratified STAD patients and was associated with immune checkpoint expression and immune infiltration. Collectively, our findings identify an OLFM4-associated gastric cancer cell state with palmitoylation-related signatures and altered metabolic activities, highlighting its potential relevance to metabolic heterogeneity in gastric adenocarcinoma.

## 1. Introduction

Gastric cancer (GC) remains one of the leading causes of cancer-related mortality worldwide and continues to represent a major global health burden despite advances in surgery, chemotherapy, targeted therapy, and immunotherapy [1,2]. Stomach adenocarcinoma (STAD), the predominant histological subtype of GC, is characterized by extensive molecular and cellular heterogeneity, which contributes to tumor progression, metastasis, therapeutic resistance, and poor clinical outcomes [3]. Therefore, a deeper understanding of intratumoral heterogeneity and its associated molecular programs is essential for identifying novel therapeutic targets and prognostic biomarkers in gastric cancer.

Previous molecular classification systems, including The Cancer Genome Atlas (TCGA) and Asian Cancer Research Group (ACRG), have significantly improved the understanding of gastric cancer biology at the transcriptomic level [4,5]. However, conventional bulk transcriptomic analyses are limited in their ability to resolve the heterogeneity of individual tumor cells within the tumor microenvironment (TME). With the development of single-cell RNA sequencing (scRNA-seq), it has become possible to characterize tumor cell populations at single-cell resolution and identify distinct malignant subtypes with specific biological features and regulatory programs [6,7]. Recent studies have further suggested that metabolic heterogeneity among malignant epithelial cells may be associated with gastric cancer progression and therapeutic response [8,9].

Altered metabolic activities are widely recognized as important features of cancer and have been associated with tumor growth, survival, and immune evasion [10]. Among diverse metabolic regulatory mechanisms, protein palmitoylation has attracted increasing attention as a reversible lipid modification that regulates protein stability, membrane localization, and signaling activity [11,12]. Dysregulation of palmitoylation-related enzymes, particularly members of the zinc finger DHHC-type (ZDHHC) family, has been implicated in the progression of multiple malignancies, including gastric cancer [12,13,14]. Nevertheless, the distribution of palmitoylation-related transcriptional signatures across specific gastric cancer cell states and their association with metabolic activities remain incompletely understood.

Olfactomedin 4 (OLFM4) is a glycoprotein frequently expressed in gastrointestinal malignancies and has been associated with tumor progression, stem-like characteristics, and poor prognosis in gastric cancer [15,16,17]. Recent studies have also suggested that OLFM4 may participate in metabolic adaptation and tumor-associated signaling pathways [18,19]. Moreover, OLFM4 is highly expressed in specific epithelial cell populations and has been implicated in maintaining cellular plasticity and stress adaptation, suggesting that it may contribute to subtype-specific biological programs in gastric cancer [20,21]. However, whether OLFM4 is associated with altered metabolic activities and palmitoylation-related signatures in specific gastric cancer cell states has not been systematically investigated.

In the present study, we integrated scRNA-seq and bulk transcriptomic data to characterize the heterogeneity of malignant epithelial cells in gastric adenocarcinoma. We identified an OLFM4-associated tumor subtype with enriched palmitoylation-related signatures and glycolytic activity. Functional validation further demonstrated that modulation of OLFM4 expression influenced the expression of ZDHHC2 and GLUT1, as well as glucose uptake and ATP production, in gastric cancer cells. In addition, we established a prognostic risk model based on subtype-associated genes and explored its relationship with immune infiltration and immune checkpoint expression. Our findings provide insight into the metabolic heterogeneity of gastric cancer and identify an OLFM4-associated gastric cancer cell state characterized by palmitoylation-related signatures and altered metabolic activities.

## 2. Materials and Methods

### 2.1. Bulk RNA-Seq Data Processing and Analysis

Bulk RNA sequencing data and corresponding clinical information of stomach adenocarcinoma (STAD) patients were obtained from The Cancer Genome Atlas (TCGA-STAD) database. A total of 403 gastric cancer samples with available transcriptomic and clinical data were included for subsequent analyses. Transcriptomic data were downloaded as TPM-normalized expression matrices and further transformed into log2(TPM + 1) values for downstream analyses. The processed bulk RNA-seq data were subsequently used for prognostic modeling, survival analysis, immune infiltration analysis, and validation of subtype-associated gene signatures. Differential expression analysis, Cox regression analysis, Kaplan–Meier survival analysis, and receiver operating characteristic (ROC) analyses were performed using R software (version 4.1.3). Statistical significance was defined as *p* < 0.05.

### 2.2. scRNA-Seq Data Processing

Single-cell RNA sequencing (scRNA-seq) data from three primary gastric cancer samples and one adjacent normal tissue sample were obtained from the GSE163558 dataset in the GEO database. Data processing was performed using the Seurat R package (version 4.0). For quality control, cells with mitochondrial gene percentages > 20%, fewer than 200 detected genes, or more than 5000 detected genes were excluded. The filtered data were normalized using the NormalizeData() function, and the top 2000 highly variable genes were identified using the FindVariableFeatures() function for downstream analyses. Batch effects among different samples were corrected using the Harmony R package. Dimensionality reduction was performed using UMAP and t-SNE, followed by Louvain clustering and cell-type annotation according to canonical marker genes. Differentially expressed genes (DEGs) among cell clusters were identified using the FindAllMarkers() function with thresholds of adjusted *p* < 0.05, log2 fold change > 0.25, and expression proportion > 0.1. The top 100 subtype-specific genes of the C3-OLFM4 subtype ranked by average log2 fold change were selected for subsequent analyses.

### 2.3. Cell Clusters Identification

Multiple groups of genes were used to identify different human cell clusters in scRNA Seq. For the epithelial cells, AGR2, PIGR, KRT19, KRT8, and KRT18 were regarded as specific markers, and SPARC, CALD1, COL1A2, COL3A1, and COL1A1 were identified as the specific markers of fibroblasts. In addition, markers for identifying endothelial cells included IGFBP5, PLVAP, STC1, COL4A1, IGFBP7, and SPARCL1. T cells were divided into 3 types, which included CD4+ T cells (CXCL13, DUSP4, ICOS, TNFRSF18, MAF, CD2, CTLA4), CD8+ T cells (GZMB, GZMA, NKG7, CCL5, GNLY) and Naïve T cells (INTS6, TSPYL2, TCF7, BCL11B, HEXIM1, IL7R). Meanwhile, we selected several markers (G0S2, FCGR3B, S100A8, CXCL8) as the specific markers for identifying neutrophil cells. Furthermore, markers of monocytes consisted of MMP12, EREG, CTSB, SPP1, and APOE. As for the plasma cells and B cells, we used two different groups of markers to distinguish them. The markers of plasma cells were IGLC3, JCHAIN, IGHA1, IGKC, and B cells specific markers were CD83, LY9, MEF2C, BANK1, MS4A1. For the classification of tumor cells, seven specific markers of gastric cancer (LGALS3, CD74, PLCG2, OLFM4, HELLS, TOP2A, GNLY) were selected for distinguishing these cells based on their expression patterns. Then, seven clusters of malignant epithelial cells were identified.

### 2.4. CNV Analysis of scRNA-Seq

With the identification of different clusters, CNV analysis was further performed in the scRNA data to estimate the malignancy level of all cells through the inferCNV. As fibroblasts and endothelial cells were annotated into reference groups, CNV scores of each epithelial cell were acquired, and cells with a score higher than the median CNV score were identified as malignant tumor cells.

### 2.5. Pseudotime Analysis of scRNA-Seq

To analyze the pseudotime of the identified malignant tumor cells, the R package Monocle2 was used to investigate their differentiation simulation. First, the Seurat object of tumor cells was transferred into the CellDataSet (CDS) object. Then, after the gene ordering had been identified, the DDRTree method was applied for both the dimensionality reduction and the trajectory inference. All parameters in the analysis were set to their default values.

### 2.6. Transcription Factor Analysis

The transcription factor (TF) analysis in malignant tumor cells was achieved through the Single-Cell Regulatory Network Inference and Clustering (SCENIC) to calculate the regulon activity score (RAS) in default characters. First, by using the GRNBoost2 algorithm implemented in pySCENIC (version 0.12.0), we built the TF genes co-expression network. Then, with the motif database, we verified the combination between these genes and TFs using RcisTarget. Furthermore, the AUCell R package was used to quantify the activity of each regulatory subset, which was regarded as the Regulon score.

### 2.7. Cell–Cell Communication Analysis

The analysis of cell–cell communication was achieved by the CellChat R package. First, we set the normalized gene expression matrix as the input to create the CellChat object. After identifying both over-expressed signaling genes and over-expressed ligand–receptor interactions, the communication probability between interacting cell groups was calculated using the R function computeCommunProb(). Furthermore, we also computed the probability of communication at the signaling pathways level with filtered communications, which was achieved by R functions including filterCommunication() and computeCommunProbPathway(). Finally, the network of cell communications was established through the R function aggregateNet(), which revealed the specific communication patterns between different cell groups.

### 2.8. Palmitoylation-Related Signature Analysis

A palmitoylation-related gene set (GOBP_PEPTIDYL_L_CYSTEINE_S_PALMITOYLATION) was obtained from the MSigDB database. The corresponding signature score was calculated using the ssGSEA algorithm implemented in the GSVA R package and used to estimate the relative enrichment of palmitoylation-related transcriptional signatures in different cell populations.

### 2.9. Immune Infiltration Analysis

To estimate immune cell infiltration and characterize the tumor immune microenvironment in the TCGA-STAD cohort, multiple computational algorithms, including ESTIMATE, ssGSEA, CIBERSORT, quanTIseq, TIMER, EPIC, and MCPcounter, were applied using the IOBR R package. These methods generated quantitative immune infiltration scores for each patient sample and were subsequently used to evaluate the association between immune infiltration patterns and the prognostic risk model.

### 2.10. Enrichment Analysis

With the method of hypergeometric distribution, enrichment analysis based on the KEGG and GO databases was applied by using the clusterProfiler R package. Significant signaling pathways were identified when the *p*-value, which was adjusted by BH, was lower than 0.05. GSVA analysis was achieved by using the fgsea R package, and the GSVA scores of each pathway in different tumor subtypes were compared to find a statistically significant result, whose significance was further quantified by the t-value.

### 2.11. The Prognosis-Based Model for Gene Screening

With the different expressed genes (DEGs) from the C3 tumor cell cluster, we chose several models based on the patients’ prognosis to perform further gene screening, which included both the univariate and multivariate Cox regression model (R function coxph() from the survival R package). As for the threshold used for filtering, the *p*-value threshold for univariate Cox regression was set to *p* < 0.1, and that for multivariate regression was set to *p* < 0.05. Then, using the timeROC R package, the model’s prediction capabilities were quantified and estimated through the area under the curve (AUC) of the time-varying receiver operating characteristic (ROC) curve.

### 2.12. The Statistical Analysis

All data were processed, analyzed, and visualized through R 4.1.3. In these steps, Pearson’s correlation analysis was applied to estimate the correlation between two continuous variables, and the Chi-square test was used to assess the correlation between categorical variables. The Wilcoxon rank-sum test or the T-test was performed for comparisons between two groups, and one-way ANOVA was applied to comparisons among groups of more than three. In the KM survival analysis, the best cut-off for separating the continuous variable was determined using the survminer R package.

### 2.13. Cell Culture

All human gastric cancer cell lines originated from the American Type Culture Collection (ATCC), which included GES-1, HGC-27, AGS, MKN45, and SGC-7901, and were cultured in a 10% fetal bovine serum (FBS) medium (DMEM or RPMI-1640) with an atmosphere of 5% CO_2_ at 37 °C. Selected cell lines were further incubated with the recombinant protein of OLFM4 (10261-OL, R&D Systems, Minneapolis, MN, USA) at a concentration of 1.5 μg/mL for 24 h prior to subsequent analyses.

### 2.14. Western Blotting

Lysates of multiple cell lines seeded in 10 cm wells were collected after cell lysis. The extracted proteins of different cell lines were separated by sodium dodecyl sulfate–polyacrylamide gel electrophoresis (SDS-PAGE) and transferred onto PVDF membranes, which were further blocked in 5% milk for 1 h at room temperature. After incubating with primary antibodies at 4 °C overnight, secondary antibodies were used to incubate membranes for 1 h at room temperature. Finally, we used the Odyssey CLx Imager for visualizing signals. Antibodies used are as follows: OLFM4 (ab85046, Abcam, Cambridge, UK), GLUT1 (ab115730, Abcam, Cambridge, UK), ZDHHC2 (sc-515204, Santa Cruz Biotechnology, Dallas, TX, USA), and β-actin (sc-69879, Santa Cruz Biotechnology, Dallas, TX, USA).

### 2.15. Small Interfering RNA Transfection

OLFM4 expression was silenced using small interfering RNA (siRNA). Cells were transfected with OLFM4-specific siRNA (si OLFM4; OriGene Technologies, Rockville, MD, USA; SR307184) or negative control siRNA (siNC) using Lipofectamine 3000 (Invitrogen, Carlsbad, CA, USA, USA) according to the manufacturer’s instructions. After transfection, knockdown efficiency was confirmed by Western blot analysis. Subsequent experiments were performed 48–72 h after transfection.

### 2.16. ATP Measurement

The treated human gastric cancer cell lines in 6-well plates were lysed and centrifuged to obtain the lysates, which were further used for measuring the ATP level according to the instructions of the ATP assay kit (HY-K0314, MedChemExpress, Monmouth Junction, NJ, USA). The expression of ATP in different samples was quantified by the strength of luminescence in the microplate reader.

### 2.17. Glucose Uptake Assay

Glucose uptake was assessed using the fluorescent glucose analog 2-NBDG (2-(N-(7-Nitrobenz-2-oxa-1,3-diazol-4-yl) amino)-2-deoxyglucose; N13195, Invitrogen, Thermo Fisher Scientific, Waltham, MA, USA). Following recombinant OLFM4 treatment or siRNA-mediated OLFM4 knockdown, AGS and HGC-27 cells were incubated with 200 μM 2-NBDG for 30 min at 37 °C. After incubation, cells were washed three times with PBS to remove excess dye and immediately imaged using a fluorescence microscope under identical exposure settings. Fluorescence intensity was quantified using ImageJ software (version 1.54g) and normalized to the corresponding control group.

## 3. Results

### 3.1. Identification and Classification of Cell Populations in scRNA-Seq Data

**Figure 1:**
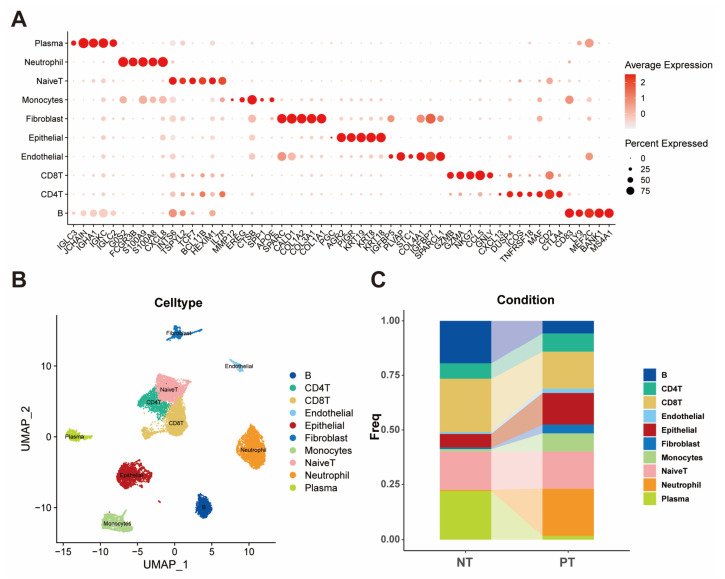
(**A**) The dot plot demonstrates specific markers for identifying different cell clusters. (**B**) The UMAP plot visualizes the classification of different cell clusters. (**C**) The bar plot represents the ratios of different cells in both the primary tumor and the normal tissue.

To investigate the cellular heterogeneity of gastric adenocarcinoma, scRNA-seq data from three primary tumor samples and one adjacent normal tissue sample in the GSE163558 cohort were analyzed. After quality control and filtering of low-quality cells, a total of 14,315 cells were retained for downstream analyses. Using unsupervised clustering and UMAP dimensional reduction, cells were classified into nine major cell populations based on the expression of established cell-type-specific markers, including B cells, CD4+ T cells, CD8+ T cells, naïve T cells, endothelial cells, epithelial cells, fibroblasts, monocytes, neutrophils, and plasma cells (Figure 1A,B). The expression patterns of representative marker genes further confirmed the accuracy of cell-type annotation. Specifically, epithelial cells highly expressed EPCAM, KRT8, and KRT19, while fibroblasts were characterized by COL1A1, COL1A2, and SPARC expression. Immune cell populations also demonstrated distinct marker profiles, including CD3D and IL7R in T-cell populations, MS4A1 and CD83 in B cells, and FCGR3B and S100A8 in neutrophils. We next compared the distribution of different cell populations between normal tissue (NT) and primary tumor tissue (PT). The proportions of epithelial cells, fibroblasts, endothelial cells, monocytes, and neutrophils increased in tumor tissues, whereas several immune-related populations showed relatively reduced proportions in normal tissues (Figure 1C).

These findings demonstrated substantial cellular heterogeneity within the gastric cancer microenvironment and provided the basis for subsequent analyses of malignant epithelial subtypes.

### 3.2. CNV Analysis Revealed Malignant Characteristics of Epithelial Cells

**Figure 2:**
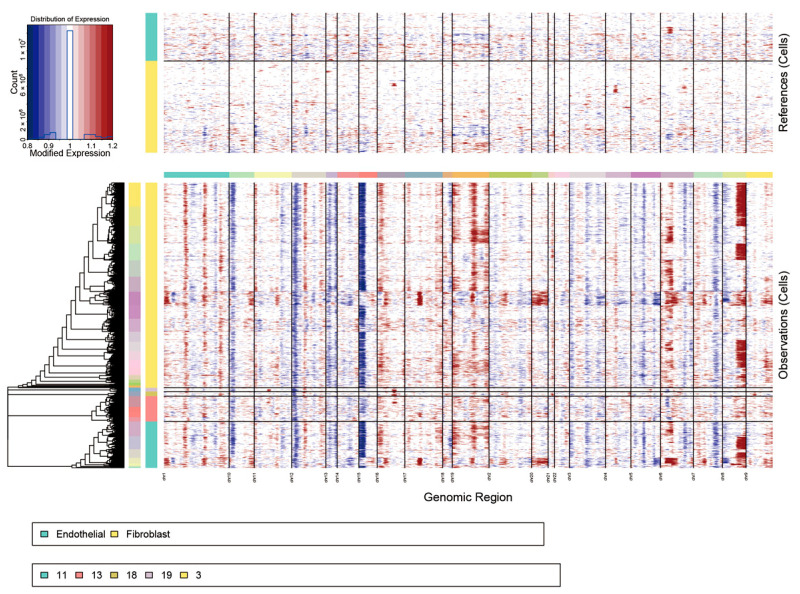
InferCNV analysis of single-cell transcriptomic data. The heatmap illustrates large-scale copy number variation (CNV) patterns across different cell populations. Fibroblasts and endothelial cells were used as reference cell populations for CNV inference. Red and blue colors indicate relative copy number gains and losses, respectively, whereas white represents neutral copy number states. Epithelial cells exhibited substantially increased CNV signals compared with reference cells, suggesting enhanced genomic instability and malignant characteristics.

To further distinguish malignant epithelial cells from non-malignant cell populations, inferCNV analysis was performed using fibroblasts and endothelial cells as reference populations. Compared with reference cells, epithelial cells exhibited substantially increased copy number variation (CNV) signals across multiple chromosomal regions, indicating enhanced genomic instability and malignant characteristics (Figure 2). In contrast, fibroblasts and endothelial cells demonstrated relatively stable CNV patterns with limited large-scale chromosomal alterations.

These findings supported the malignant nature of epithelial cell populations and provided the basis for subsequent analyses of tumor cell heterogeneity in gastric adenocarcinoma.

### 3.3. Heterogeneity of Malignant Epithelial Cells in STAD

**Figure 3:**
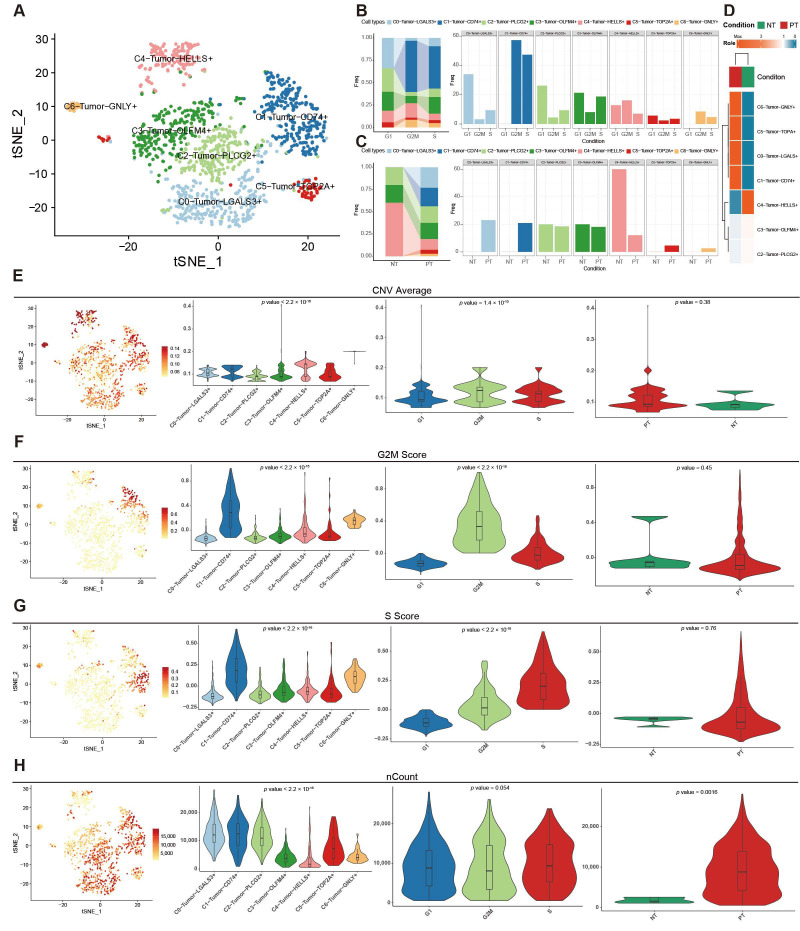
(**A**) tSNE plot demonstrates seven subtypes of malignant epithelial cells. (**B**,**C**) Bar plots present the ratio of each subtype in different cell cycle phases and tissue types. (**D**) Heatmap of Ro/e analysis shows the enrichment of different tumor subtypes in different tissue groups. (**E**–**H**) tSNE plots and violin plots reveal the diversity of multiple tumor subtypes in different dimensions.

To further characterize the heterogeneity of malignant epithelial cells, tumor cells were classified into seven distinct subtypes based on subtype-specific marker expression, including LGALS3, CD74, PLCG2, OLFM4, HELLS, TOP2A, and GNLY (Figure 3A). These tumor subtypes were designated as C0 to C6 according to their representative markers. Cell-cycle analysis revealed substantial differences among tumor subtypes. The C1-CD74 subtype demonstrated relatively high G2M and S phase activity, suggesting enhanced proliferative potential, whereas several other subtypes showed relatively lower cell-cycle activity (Figure 3B). In addition, comparison of tissue origins demonstrated that C0, C1, C5, and C6 were predominantly enriched in primary tumor tissues, while the C4-HELLS subtype was mainly distributed in normal tissues (Figure 3C,D). Further analyses of CNV scores, G2M scores, S scores, and transcript counts also demonstrated significant heterogeneity among different tumor subtypes in terms of genomic instability, proliferative status, and transcriptional activity (Figure 3E–H). Notably, the C3-OLFM4 subtype exhibited intermediate levels of malignant characteristics and cell-cycle activity compared with other tumor subtypes.

Collectively, these findings demonstrated substantial intratumoral heterogeneity among malignant epithelial cell populations in gastric adenocarcinoma and suggested the presence of distinct biological states within tumor cells.

### 3.4. Cell–Cell Communication Analysis Revealed Distinct Signaling Interactions Among Tumor Subtypes

**Figure 4:**
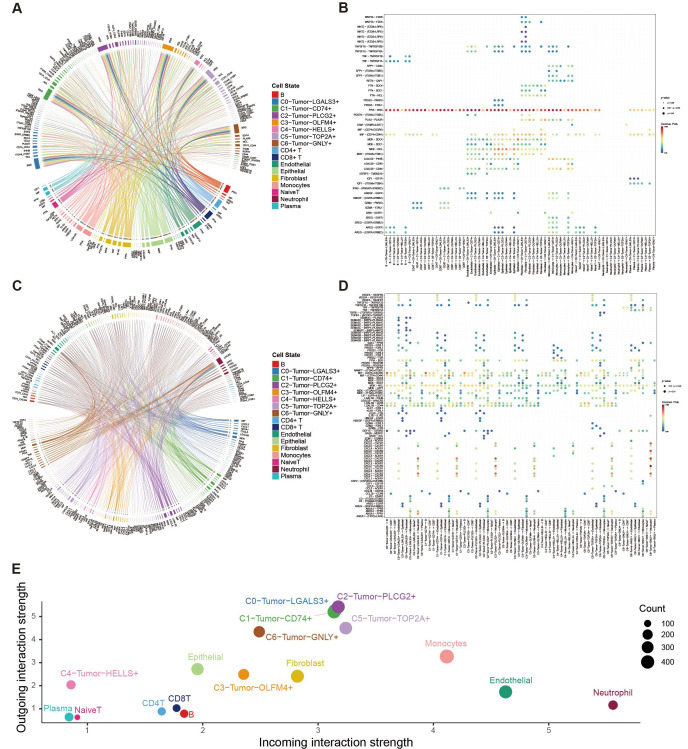
(**A**,**B**) The chord graph and the dot plot demonstrate the incoming interaction of tumor cells with other cells and the corresponding ligand–receptor pairs. (**C**,**D**) The chord graph and the dot plot demonstrate the outgoing interaction of tumor cells with other cells and the corresponding ligand–receptor pairs. (**E**) The dot plot presents both the incoming and outgoing cell–cell interaction strength of all cell types.

To further investigate intercellular communication within the gastric cancer microenvironment, CellChat analysis was performed to evaluate ligand–receptor interactions among different cell populations. Incoming signaling analysis revealed extensive interactions between tumor cells and surrounding stromal or immune cell populations, with the PPIA–BSG signaling axis representing one of the dominant interaction patterns (Figure 4A,B). In contrast, outgoing signaling activities varied substantially among tumor subtypes. The C0-LGALS3, C1-CD74, C2-PLCG2, and C5-TOP2A subtypes exhibited relatively stronger outgoing communication capacities compared with other tumor subtypes (Figure 4C,D). Several signaling pathways, including MDK–NCL and MIF–CD74/CD44 interactions, were enriched in these actively communicating tumor populations. Quantitative comparison of interaction strengths further demonstrated distinct communication patterns among different tumor subtypes and microenvironmental cell populations (Figure 4E). Notably, the C3-OLFM4 subtype exhibited moderate incoming and outgoing interaction activities relative to other malignant subtypes.

These findings suggested substantial heterogeneity in cell–cell communication networks among gastric cancer tumor subtypes and highlighted distinct signaling states within the tumor microenvironment.

### 3.5. Cellular Differentiation Analysis Revealed Distinct Developmental States Among Tumor Subtypes

**Figure 5:**
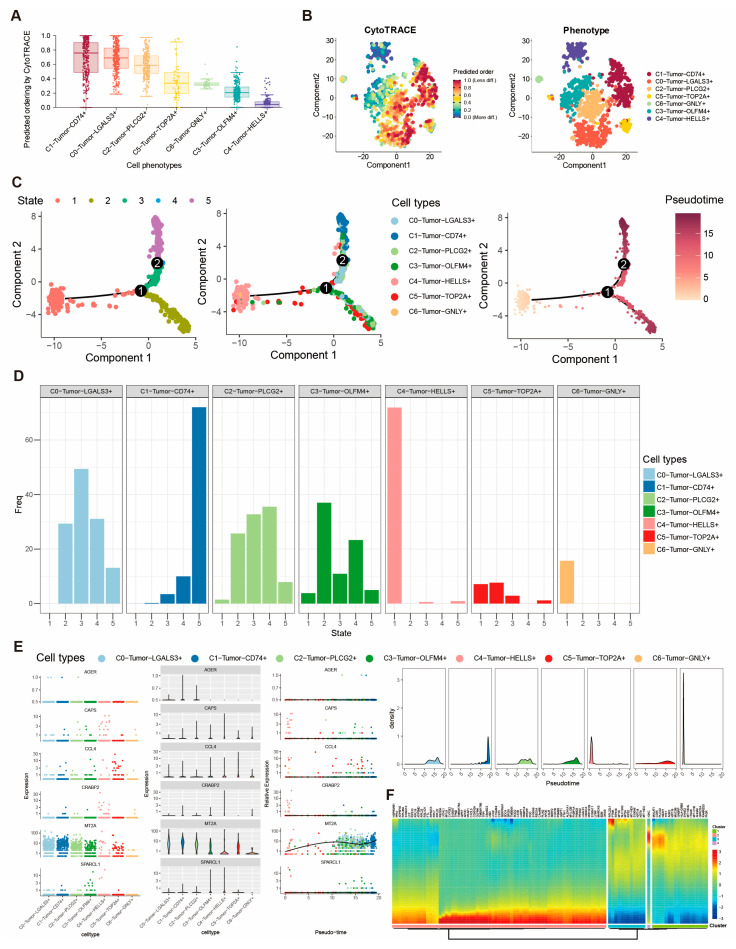
(**A**,**B**) Box plot and tSNE plot were used to visualize and compare the CytoTRACE score of each tumor subtype, representing their cellular plasticity. (**C**) Plots of the trajectory analysis demonstrate different states and the differentiation trajectory according to the pseudotime analysis. (**D**) The bar plot shows the distribution of seven tumor subtype cells in 5 differentiation states. (**E**,**F**) Expression of differentiation-related genes was visualized based on the pseudotime trajectory.

To further characterize the functional heterogeneity of malignant epithelial cells, CytoTRACE analysis was performed to estimate cellular differentiation potential among different tumor subtypes. The C1-CD74 subtype exhibited the highest CytoTRACE scores, suggesting a relatively less differentiated cellular state, whereas the C4-HELLS subtype demonstrated relatively low differentiation potential (Figure 5A,B). Pseudotime trajectory analysis further classified tumor cells into five differentiation states distributed along distinct developmental trajectories (Figure 5C). Different tumor subtypes showed heterogeneous distributions across these differentiation states. Notably, the C1-CD74 subtype was predominantly enriched in advanced differentiation states, whereas the C4-HELLS subtype was mainly restricted to early states (Figure 5D). To further investigate differentiation-associated transcriptional changes, several differentiation-related genes, including AGER, CAPS, CCL4, CRABP2, MT2A, and SPARCL1, were analyzed across pseudotime trajectories (Figure 5E). Among these genes, MT2A exhibited a gradual increase along pseudotime progression, suggesting its potential association with tumor cell differentiation. Additional pseudotime-associated genes were further identified and visualized (Figure 5F).

These findings demonstrated substantial differentiation heterogeneity among malignant epithelial subtypes and suggested that distinct tumor populations may exist in different developmental and functional states within gastric adenocarcinoma.

### 3.6. OLFM4-Associated Tumor Cells Exhibited Enriched Palmitoylation-Related Signatures and Altered Metabolic Activities

**Figure 6:**
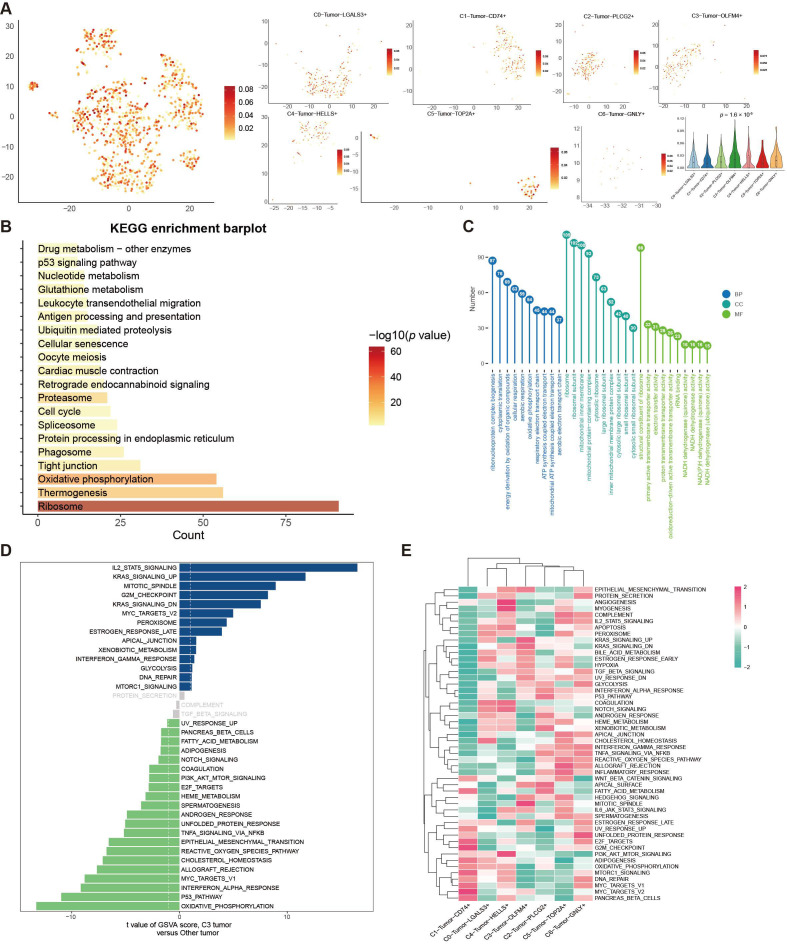
(**A**) Feature plots and violin plots demonstrate the palmitoylation-related signature scores among different tumor subtypes. (**B**,**C**) KEGG and GO enrichment analyses of differentially expressed genes in the C3-OLFM4 subtype. KEGG pathways were ranked according to the −log10 (*p*-value), while GO terms were ranked by gene counts. (**D**) GSVA analysis shows the t-values of pathway activities in the C3-OLFM4 subtype compared with other tumor subtypes. (**E**) Heatmap visualizes the GSVA scores of multiple signaling pathways across different tumor subtypes.

**Figure 7:**
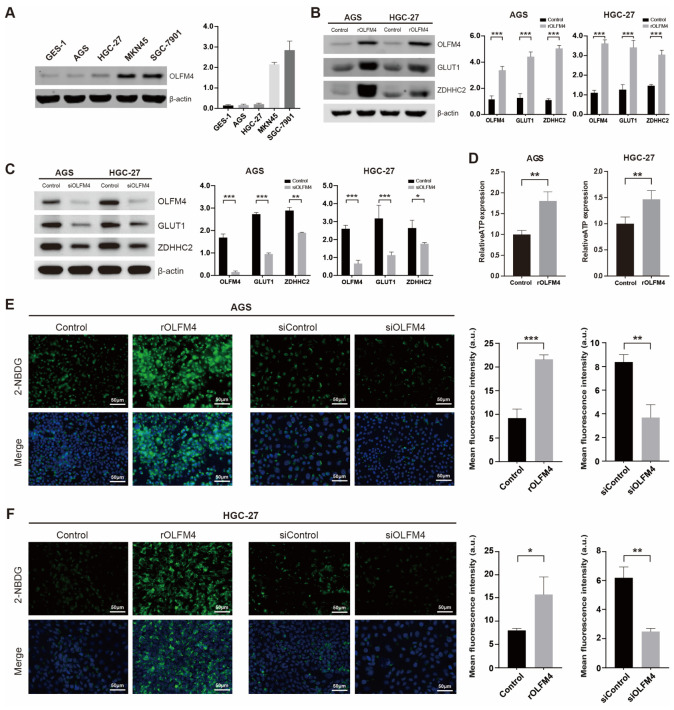
(**A**) Western blot analysis of endogenous OLFM4 expression in five gastric cell lines (GES-1, AGS, HGC-27, MKN45, and SGC-7901). (**B**) Western blot analysis and corresponding quantification of OLFM4, GLUT1, and ZDHHC2 expression in AGS and HGC-27 cells following recombinant OLFM4 treatment. (**C**) Western blot analysis and corresponding quantification of OLFM4, GLUT1, and ZDHHC2 expression in AGS and HGC-27 cells following siRNA-mediated OLFM4 knockdown. (**D**) ATP production assay in AGS and HGC-27 cells following recombinant OLFM4 treatment. (**E**,**F**) Representative fluorescence images and quantitative analysis of 2-NBDG uptake in AGS (**E**) and HGC-27 (**F**) cells under recombinant OLFM4 treatment or siRNA-mediated OLFM4 knockdown. Scale bars = 50 μm. Data are presented as mean ± SD. * *p* < 0.05, ** *p* < 0.01, *** *p* < 0.001. The original images of Western Blots can be found in the Supplementary Materials.

To further investigate subtype-specific biological characteristics, pathway enrichment analyses were performed among different tumor subtypes. Palmitoylation-related signature analysis revealed that the C3-OLFM4 subtype exhibited the highest palmitoylation-related signature scores among all tumor subtypes (Figure 6A). Consistently, KEGG and GO enrichment analyses demonstrated significant enrichment of multiple metabolism-related pathways, including oxidative phosphorylation, thermogenesis, and ATP metabolic processes in the C3 subtype (Figure 6B,C). To further characterize pathway activity, GSVA analysis was performed across all tumor subtypes. The C3-OLFM4 subtype demonstrated significant enrichment of multiple metabolic pathways, particularly glycolysis-related signaling and oxidative phosphorylation (Figure 6D,E), suggesting altered metabolic activities in this tumor population. To further evaluate the association between OLFM4 expression and metabolic activities, gastric cancer cell lines with relatively low endogenous OLFM4 expression, including AGS and HGC-27, were selected for functional experiments (Figure 7A). Recombinant OLFM4 treatment increased the expression of ZDHHC2 and the glycolytic transporter GLUT1 in both AGS and HGC-27 cells (Figure 7B). Conversely, siRNA-mediated OLFM4 knockdown reduced the expression of both ZDHHC2 and GLUT1 in AGS and HGC-27 cells (Figure 7C). To further evaluate glucose metabolic activity, 2-NBDG uptake assays were performed in AGS and HGC-27 cells. Recombinant OLFM4 treatment significantly increased 2-NBDG fluorescence intensity in both cell lines, whereas OLFM4 silencing markedly reduced glucose uptake capacity, as indicated by decreased 2-NBDG fluorescence signals (Figure 7E,F). Consistent with the enhanced glucose uptake phenotype, recombinant OLFM4 treatment significantly increased ATP production in AGS and HGC-27 cells (Figure 7D).

Collectively, these findings suggested that the OLFM4-associated tumor subtype was characterized by enriched palmitoylation-related signatures and altered metabolic activities. Functional experiments further demonstrated an association between OLFM4 expression and glucose metabolic activity in gastric cancer cells.

### 3.7. Transcription Factor Analysis Revealed Distinct Regulatory Programs Among Tumor Subtypes

**Figure 8:**
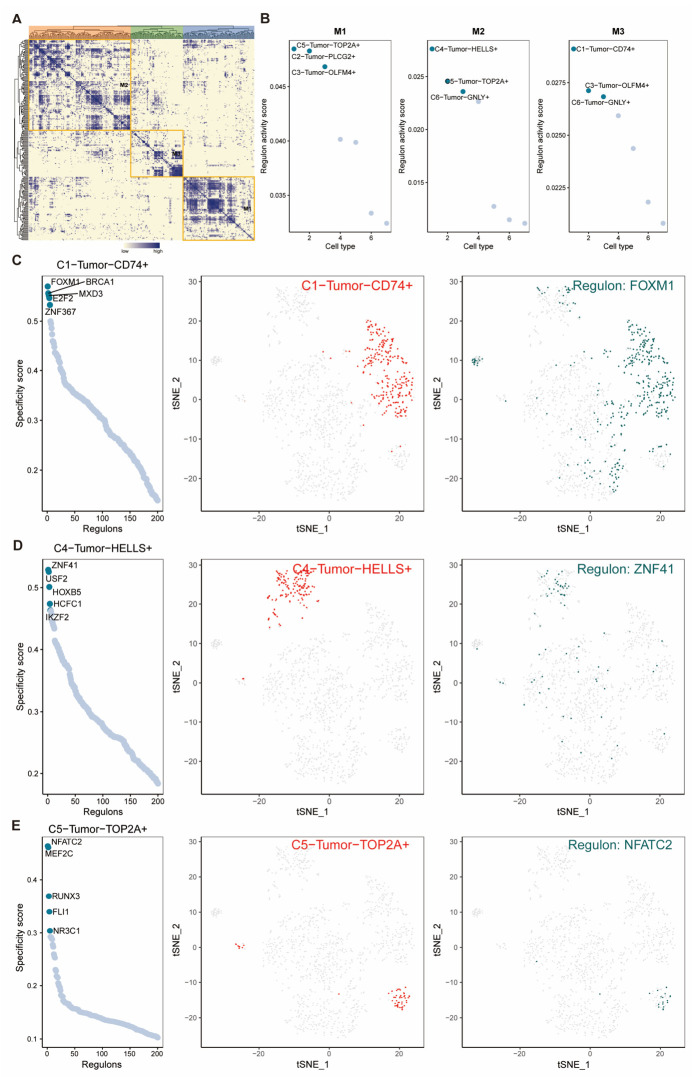
(**A**) Heatmap of Regulon CSI matrix demonstrates the classification of TFs, which classified tumor cells into 3 groups (M1, M2, M3). (**B**) Dot plots show average regulon activity scores of different tumor subtypes in each module and label the significantly related tumor subtypes. (**C**–**E**) Dot plots (**left**) present the rank for regulons according to the regulon specificity score (RSS) in each tumor subtype, and the tSNE plots reveal the specific tumor subtype (**middle**, red dots) and the top-ranked regulon (**right**, cyan dots) with the highest specificity scores in corresponding tumor subtypes.

To further investigate transcriptional regulatory heterogeneity among malignant epithelial subtypes, pySCENIC analysis was performed to identify subtype-specific transcription factor (TF) regulons. Based on the connection specificity index (CSI) of regulon activities, TFs were classified into three major regulatory modules, designated as M1, M2, and M3 (Figure 8A). Different tumor subtypes exhibited distinct regulon activity patterns across these modules (Figure 8B). The M1 module was primarily associated with the C2-PLCG2, C3-OLFM4, and C5-TOP2A subtypes, whereas the M2 module was enriched in the C4-HELLS, C5-TOP2A, and C6-GNLY subtypes. In contrast, the M3 module showed relatively higher activity in the C1-CD74, C3-OLFM4, and C6-GNLY subtypes. To further characterize subtype-specific regulatory programs, representative regulons with high specificity scores were analyzed in different tumor subtypes. FOXM1 regulon activity was predominantly enriched in the C1-CD74 subtype (Figure 8C), whereas ZNF41 and NFATC2 regulons showed relatively specific enrichment in the C4-HELLS and C5-TOP2A subtypes, respectively (Figure 8D,E).

These findings demonstrated substantial transcriptional regulatory heterogeneity among malignant epithelial subtypes and suggested the existence of distinct subtype-associated transcriptional programs in gastric adenocarcinoma.

### 3.8. Construction of a Prognostic Risk Model Associated with the C3-OLFM4 Subtype

**Figure 9:**
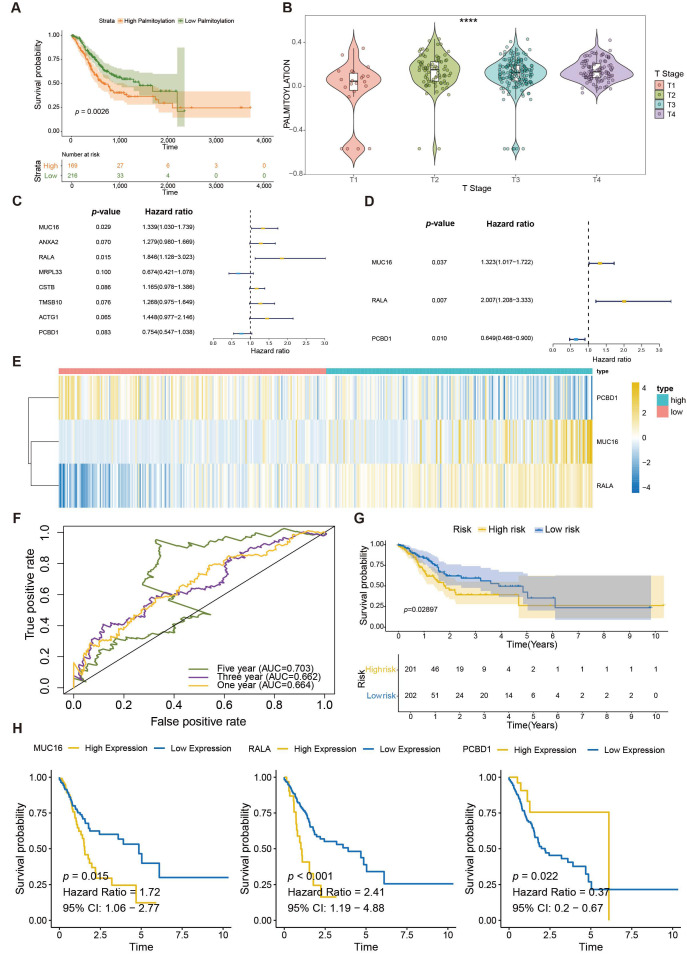
(**A**) Kaplan–Meier survival analysis according to palmitoylation-related signature scores in the TCGA-STAD cohort. (**B**) The violin plot presents the activity level of palmitoylation in different T stages. (**C**,**D**) Forest plots present the results of *p*-value, hazard ratios, and 95% confidence interval (CI) for genes in univariate and multivariate Cox analyses in the TCGA-STAD cohort. The *p*-value cut-off was *p* < 0.1 and *p* < 0.05 in the univariate and multivariate Cox analyses, respectively. The hazard ratio (HR) > 1 was identified as the reverse effect on survival and <1 was regarded as the protective effect on survival. (**E**) Heatmap demonstrates the expression of 3 genes in high/low risk groups of TCGA-STAD, with the median of the risk score set as the cut-off. (**F**) ROC curves of survival outcomes indicate the predictive performance of the risk-score model. (**G**,**H**) KM survival plots show the significant relationship between the overall survival (OS) and not only the risk groups (**G**) but also the risk-score-related genes (MUC16, RALA, PCBD1) (**H**). *p* < 0.05 was identified as statistically significant, **** *p* < 0.0001.

To further investigate the clinical significance of the C3-OLFM4 subtype, palmitoylation-related signature scores were analyzed in the TCGA-STAD cohort. Higher palmitoylation-related signature scores were significantly associated with poorer overall survival and advanced T stage in gastric cancer patients (Figure 9A,B). To establish a prognostic model associated with the C3 subtype, the top 100 subtype-specific genes ranked by average log2 fold change were subjected to Cox regression analyses. Univariate Cox analysis identified eight prognosis-associated genes, including MUC16, ANXA2, RALA, MRPL33, CSTB, TMSB10, ACTG1, and PCBD1 (Figure 9C). Further multivariate Cox analysis identified three independent prognostic genes, consisting of MUC16, RALA, and PCBD1 (Figure 9D). Based on these three genes, a prognostic risk model was established to stratify TCGA-STAD patients into high-risk and low-risk groups. Expression heatmaps demonstrated distinct expression patterns of these genes between the two groups (Figure 9E). Receiver operating characteristic (ROC) analysis showed moderate predictive performance of the risk model, with AUC values of 0.664, 0.662, and 0.703 for 1-, 3-, and 5-year survival, respectively (Figure 9F). Kaplan–Meier survival analysis further demonstrated significantly poorer overall survival in the high-risk group compared with the low-risk group (Figure 9G). In addition, survival analyses of individual model genes revealed that high expression of MUC16 and RALA was associated with unfavorable prognosis, whereas high PCBD1 expression correlated with improved survival outcomes (Figure 9H).

These findings suggested that the C3-OLFM4-associated risk model possessed prognostic value and effectively stratified patients with gastric adenocarcinoma into distinct risk groups.

### 3.9. Correlations Between Immune Infiltration and the Risk Score

**Figure 10:**
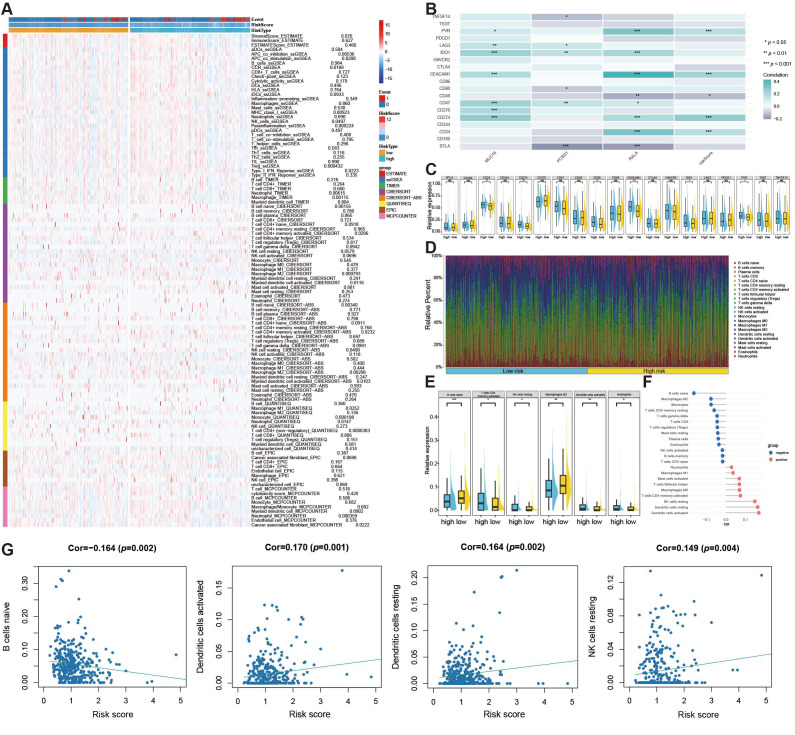
(**A**) The heatmap presents the quantified immune infiltration of the TCGA-STAD cohort based on multiple immune-related gene sets. (**B**,**C**) Heatmap and boxplots showing the expression of immune checkpoint molecules associated with the risk score. *p* < 0.05 was identified as statistically significant. (**D**) The bar plot demonstrates the relative percentage of different immune cells according to the CIBERSORT analysis. (**E**) Boxplots indicate immune cells with different infiltration between high- and low-risk groups. (**F**,**G**) The bar plot and dot plots show immune cells that were significantly correlated with risk scores (*p* < 0.05).

To further investigate the immunological characteristics associated with the C3-OLFM4-related risk model, multiple computational algorithms, including ESTIMATE, ssGSEA, CIBERSORT, quanTIseq, TIMER, EPIC, and MCPcounter, were applied to evaluate immune infiltration in the TCGA-STAD cohort (Figure 10A). Distinct immune infiltration patterns were observed between high-risk and low-risk groups. Correlation analyses further demonstrated significant associations between the risk model and multiple immune checkpoint molecules, including CD274 (PD-L1), IDO1, CEACAM1, and PVR (Figure 10B,C), suggesting a potential relationship between the C3-OLFM4-associated subtype and the immunosuppressive tumor microenvironment. CIBERSORT analysis revealed differences in the relative proportions of immune cell populations between high-risk and low-risk groups (Figure 10D). Several immune cell types, including naïve B cells, activated CD4 memory T cells, resting NK cells, M2 macrophages, activated dendritic cells, and neutrophils, showed significant differences between the two groups (Figure 10E). Correlation analysis between immune cell infiltration and risk scores further identified significant associations with naïve B cells, activated dendritic cells, resting dendritic cells, and resting NK cells (Figure 10F,G).

These findings suggested that the C3-OLFM4-associated risk model was closely associated with immune infiltration and immune checkpoint expression in gastric adenocarcinoma.

## 4. Discussion

In the present study, we integrated single-cell and bulk transcriptomic analyses to investigate the heterogeneity of malignant epithelial cells in gastric adenocarcinoma. We identified seven distinct tumor subtypes with diverse biological characteristics and further demonstrated that the OLFM4-associated C3 subtype exhibited enriched palmitoylation-related signatures and altered metabolic activities. Functional validation further showed that modulation of OLFM4 expression influenced the expression of ZDHHC2 and GLUT1 in gastric cancer cells, accompanied by altered glucose uptake and ATP production. In addition, we established a prognostic risk model associated with the C3 subtype and demonstrated its relationship with immune infiltration and immune checkpoint expression. Collectively, these findings suggest that OLFM4-associated tumor cells may represent a distinct gastric cancer cell state with altered metabolic features.

Interestingly, the C3-OLFM4 subtype did not exhibit the highest proliferative activity or genomic instability compared with other tumor subtypes, but instead demonstrated relatively intermediate malignant characteristics accompanied by enhanced metabolic features and enriched palmitoylation-related signatures. This observation suggests that metabolic heterogeneity in gastric adenocarcinoma may not be exclusively dependent on proliferative status or chromosomal instability. Rather, distinct tumor populations may exhibit different metabolic features during tumor progression. Such findings further support the importance of subtype-specific altered metabolic features in gastric cancer heterogeneity [22,23,24].

Tumor heterogeneity is widely recognized as a major contributor to tumor progression, therapeutic resistance, and clinical outcome in gastric cancer [25,26]. Although previous molecular classification systems, including TCGA and ACRG, have improved the understanding of gastric cancer at the transcriptomic level, they remain limited in resolving the functional diversity of individual tumor cells within the tumor microenvironment [27,28]. With the development of scRNA-seq technology, it has become possible to characterize malignant epithelial populations at single-cell resolution and identify subtype-specific biological programs [29,30]. In our study, distinct tumor subtypes demonstrated substantial differences in CNV status, proliferative activity, differentiation state, transcriptional regulation, and cell–cell communication patterns, further highlighting the complexity of gastric cancer heterogeneity.

Among these tumor subtypes, the C3-OLFM4 subtype attracted our attention because of its enrichment in palmitoylation-related and metabolic pathways. OLFM4 has been reported to participate in gastric tumorigenesis and has been associated with stem-like characteristics, intestinal metaplasia, and unfavorable prognosis in gastric cancer [31,32,33]. However, its association with metabolic activities in gastric cancer remains incompletely understood. In the present study, enrichment analyses demonstrated that the C3 subtype exhibited increased glycolysis-related signaling, oxidative phosphorylation, and ATP metabolic processes, suggesting altered metabolic activities in this tumor population. These observations are consistent with previous studies reporting that altered metabolic activities are frequently associated with malignant progression [10,34].

Protein palmitoylation has emerged as an important post-translational modification involved in protein stability, membrane localization, and intracellular signaling [14,35,36]. Dysregulation of palmitoylation-associated enzymes, particularly members of the ZDHHC family, has been implicated in multiple malignancies, including gastric cancer [37,38]. In our study, the C3-OLFM4 subtype demonstrated the highest palmitoylation-related signature scores among all tumor subtypes. Furthermore, recombinant OLFM4 treatment increased the expression of the palmitoylation-associated marker ZDHHC2 and the glycolytic transporter GLUT1 in gastric cancer cells, whereas siRNA-mediated OLFM4 knockdown reduced their expression. These findings suggest a potential association between OLFM4 expression, palmitoylation-related signatures, and altered metabolic features in gastric cancer cells. However, direct measurements of protein palmitoylation were not performed in the present study. Therefore, the observed findings should be interpreted as associations with palmitoylation-related transcriptional signatures rather than definitive evidence of altered palmitoylation activity.

Altered metabolic activities are closely associated with tumor growth and adaptation to stress conditions within the tumor microenvironment [39]. Glycolysis-related pathways were significantly enriched in the C3 subtype, and OLFM4 modulation altered GLUT1 expression, glucose uptake, and ATP production in gastric cancer cells. GLUT1 is a critical glucose transporter involved in glucose uptake and glycolytic metabolism and has been reported to contribute to malignant progression in gastric cancer [39,40,41]. Our findings suggest that OLFM4-associated tumor cells may exhibit increased glucose uptake and ATP production, together with altered metabolic features. Nevertheless, additional studies, including metabolic flux analyses and palmitoylation-specific assays, will be necessary to further clarify the underlying mechanisms.

In addition to metabolic characteristics, we further established a prognostic risk model based on three C3 subtype-associated genes, including MUC16, RALA, and PCBD1. This model demonstrated moderate predictive performance and successfully stratified gastric cancer patients into different prognostic groups. Previous studies have reported that MUC16 and RALA are associated with tumor progression and immune regulation in several malignancies [42,43,44]. In contrast, the role of PCBD1 in gastric cancer remains relatively unclear. Notably, although OLFM4 served as the defining marker of the C3 subtype and was therefore selected for subsequent functional validation, it was not retained in the final prognostic model. This observation highlights that subtype-defining markers and prognostic biomarkers may represent distinct biological dimensions. The three-gene signature was selected through multivariate survival analyses based on prognostic performance rather than biological prioritization of individual subtype markers. Moreover, the risk model was significantly associated with immune checkpoint expression and immune infiltration patterns, suggesting a potential relationship between altered metabolic activities and the immunological tumor microenvironment. These findings are consistent with previous studies reporting interactions between tumor metabolism and immune regulation in gastric cancer. At present, our data do not support a direct mechanistic relationship between OLFM4 and the three prognostic genes, and further studies will be required to clarify their potential biological connections.

Several limitations should also be acknowledged in the present study. First, the number of scRNA-seq samples included in this study was relatively limited and may not fully capture the entire spectrum of gastric cancer heterogeneity. Second, although functional experiments demonstrated that modulation of OLFM4 expression influenced ZDHHC2 and GLUT1 expression, glucose uptake, and ATP production, the relationship between OLFM4 expression, palmitoylation-related signatures, and altered metabolic activities remains insufficiently characterized. Additional in vitro and in vivo studies will be necessary to validate these observations and further investigate the underlying signaling pathways. Third, the prognostic risk model was established using the TCGA-STAD cohort and still requires validation in independent external cohorts before clinical application.

In summary, our study identified an OLFM4-associated tumor subtype characterized by enriched palmitoylation-related signatures and altered metabolic activities in gastric adenocarcinoma. Functional validation further supported an association between OLFM4 expression, glucose uptake, and ATP production in gastric cancer cells. These findings provide additional insight into the metabolic heterogeneity of gastric cancer and may contribute to future investigations of subtype-specific therapeutic strategies.

## 5. Conclusions

In summary, this study identified a distinct OLFM4-associated gastric cancer cell state in gastric adenocarcinoma characterized by enriched palmitoylation-related signatures and altered metabolic activities. Functional validation further supported an association between OLFM4 expression, GLUT1 and ZDHHC2 expression, glucose uptake, and ATP production in gastric cancer cells. Furthermore, a prognostic risk model derived from C3-subtype-associated genes effectively stratified patient outcomes and was associated with immune infiltration patterns and immune checkpoint expression. These findings provide additional insight into the metabolic heterogeneity of gastric adenocarcinoma and support a potential association between OLFM4 expression and subtype-specific metabolic features, which warrants further mechanistic investigation.

## Data Availability

All datasets analyzed in this study are publicly available. The bulk RNA sequencing data of stomach adenocarcinoma (STAD) were obtained from The Cancer Genome Atlas (TCGA-STAD) (https://portal.gdc.cancer.gov/, accessed on 21 June 2025), and the scRNA sequencing dataset was downloaded from the Gene Expression Omnibus (GEO) under accession number GSE163558 (https://www.ncbi.nlm.nih.gov/geo/query/acc.cgi?acc=GSE163558, accessed on 21 June 2025). Additional processed data supporting the findings of this study, including normalized expression matrices, differential expression results, and prognostic model coefficients, are available from the corresponding author upon reasonable request.

## Supplementary Materials

The following supporting information can be downloaded at: https://www.mdpi.com/article/10.3390/biom16060880/s1. The original Western blot images can be found in the supplementary materials.

## Abbreviations

The following abbreviations are used in this manuscript:

OLFM4	Olfactomedin 4
GC	Gastric Cancer
scRNA	Single-Cell RNA Sequencing
STAD	Stomach Adenocarcinoma
TME	Tumor Microenvironment
TCGA	The Cancer Genome Atlas
CAFs	Cancer-Associated Fibroblasts
HK2	Hexokinase 2
VDACs	Voltage-Dependent Anion Channels
ZDHHC2	Zinc Finger DHHC-Type Palmitoyltransferase 2
GLUT1	Glucose Transporter 1
ATP	Adenosine Triphosphate
TF	Transcription Factor
RAS	Regulon Activity Scores
GSVA	Gene Set Variation Analysis
KEGG	Kyoto Encyclopedia of Genes and Genomes
GO	Gene Ontology
GSEA	Gene Set Enrichment Analysis
DEGs	Different Expressed Genes
AUC	Area Under the Curve
ATCC	American Type Culture Collection
FBS	Fetal Bovine Serum
PVDF	Polyvinylidene Fluoride
CNV	Copy Number Variation
PT	Primary Tumor tissue
NT	Normal Tissue
MUC16	Mucin 16
RALA	RAS-Like Proto-Oncogene A
PCBD1	Pterin-4 Alpha-Carbinolamine Dehydratase 1
MYC	Myelocytomatosis
ROC	Receiver Operating Characteristic
KM	Kaplan–Meier
OS	Overall Survival
CI	Confidence Interval
HR	Hazard Ratio
LNM	Lymph Node Metastasis
