# Supplementary material for: Single-Cell Transcriptomic Analysis Identifies an OLFM4-Associated Gastric Cancer Cell State with Palmitoylation-Related Signatures and Altered Metabolic Activities
Source: Biomolecules. 2026 Jun 15;16(6):880. doi: 10.3390/biom16060880 (PMC13296650; doi:10.3390/biom16060880)

Figure 6. (F) The protein expression level of OLFM4 in GC cell lines was detected by Western blot (n = 3).

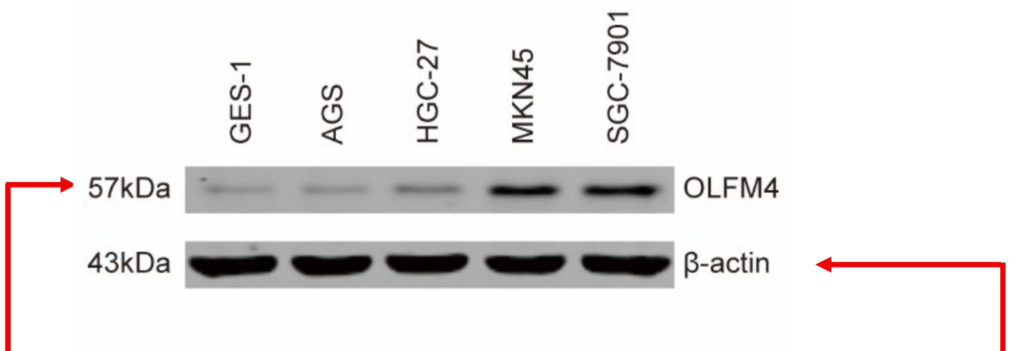

Sample 1

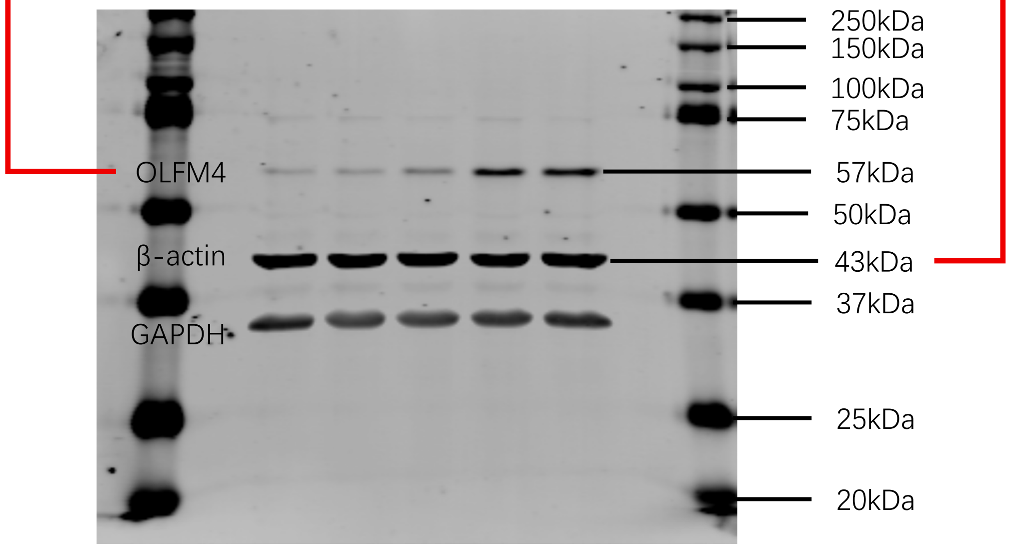

Precision Plus Protein™ Standards, #161-0374, BIO-RAD

Figure 6. (F) The protein expression level of OLFM4 in GC was detected by Western blot (n = 3).

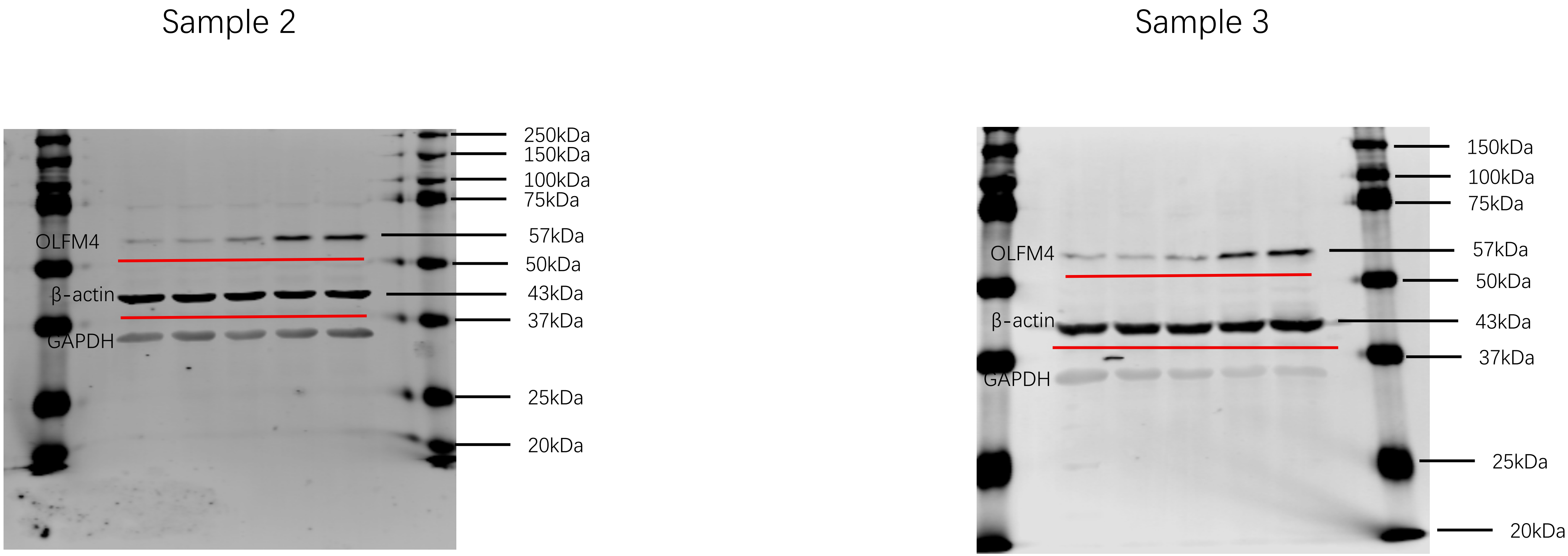

Precision Plus Protein™ Standards, #161-0374, BIO-RAD

Figure 6. (G) The protein expression level of OLFM4, GLUT1, ZDHHC2, and β-actin in the recombinant-protein treated GC cell lines was detected by Western blot (n = 3).

Sample 1

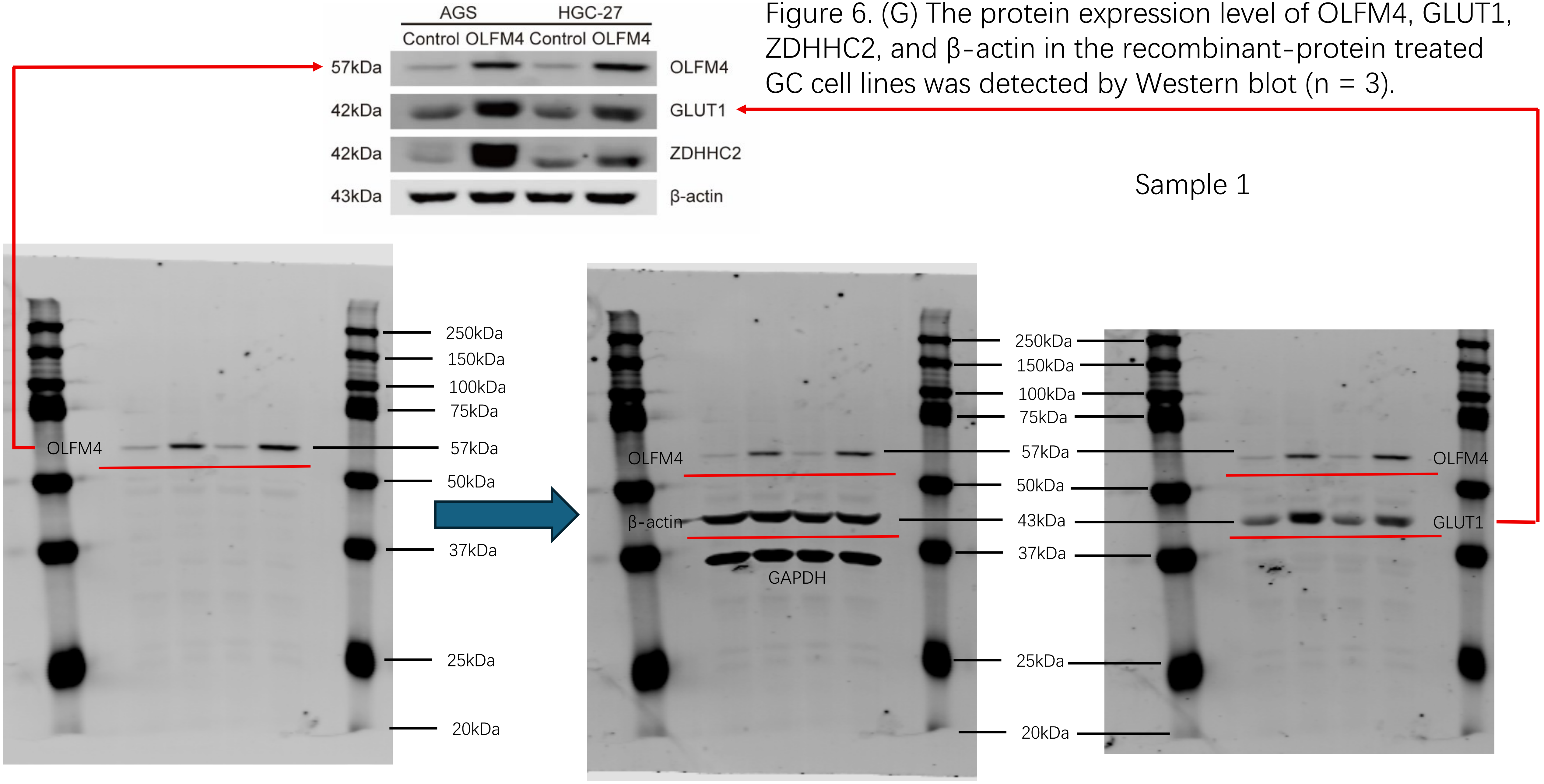

Precision Plus Protein™ Standards, #161-0374, BIO-RAD

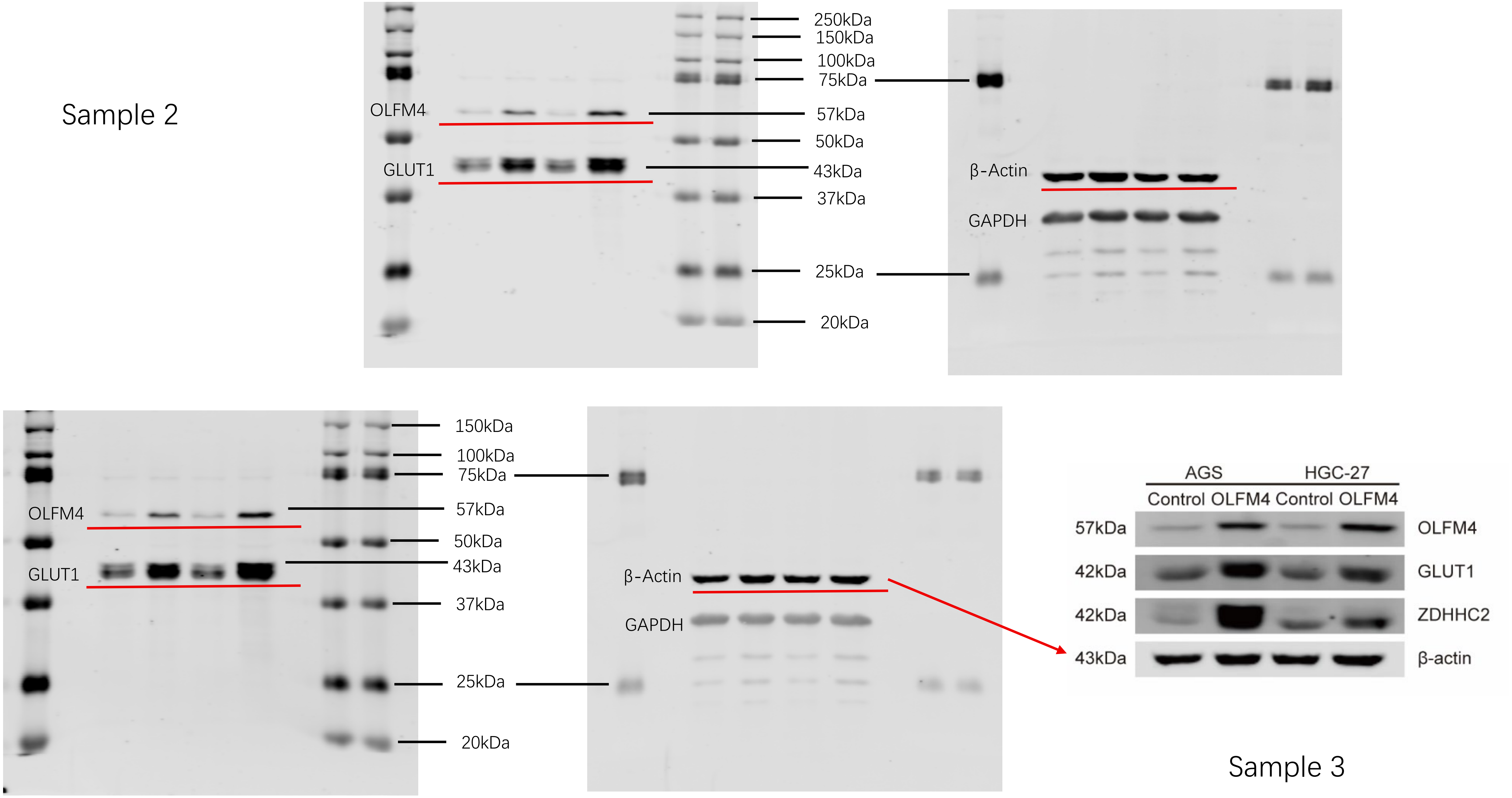

Precision Plus Protein™ Standards, #161-0374, BIO-RAD

Sample 1

Figure 6. (G) The protein expression level of OLFM4, GLUT1, ZDHHC2, and β-actin in the recombinant-protein treated GC cell lines was detected by Western blot (n = 3).

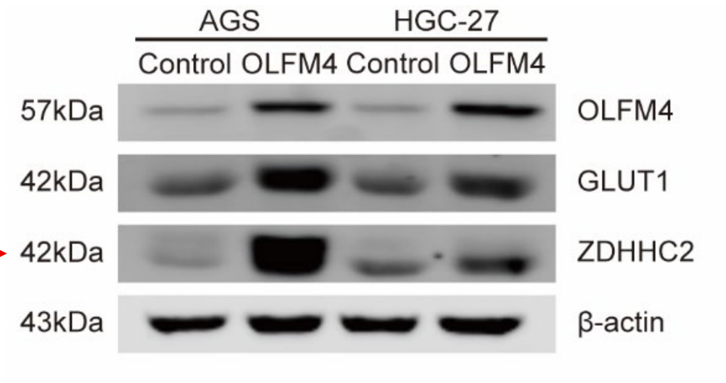

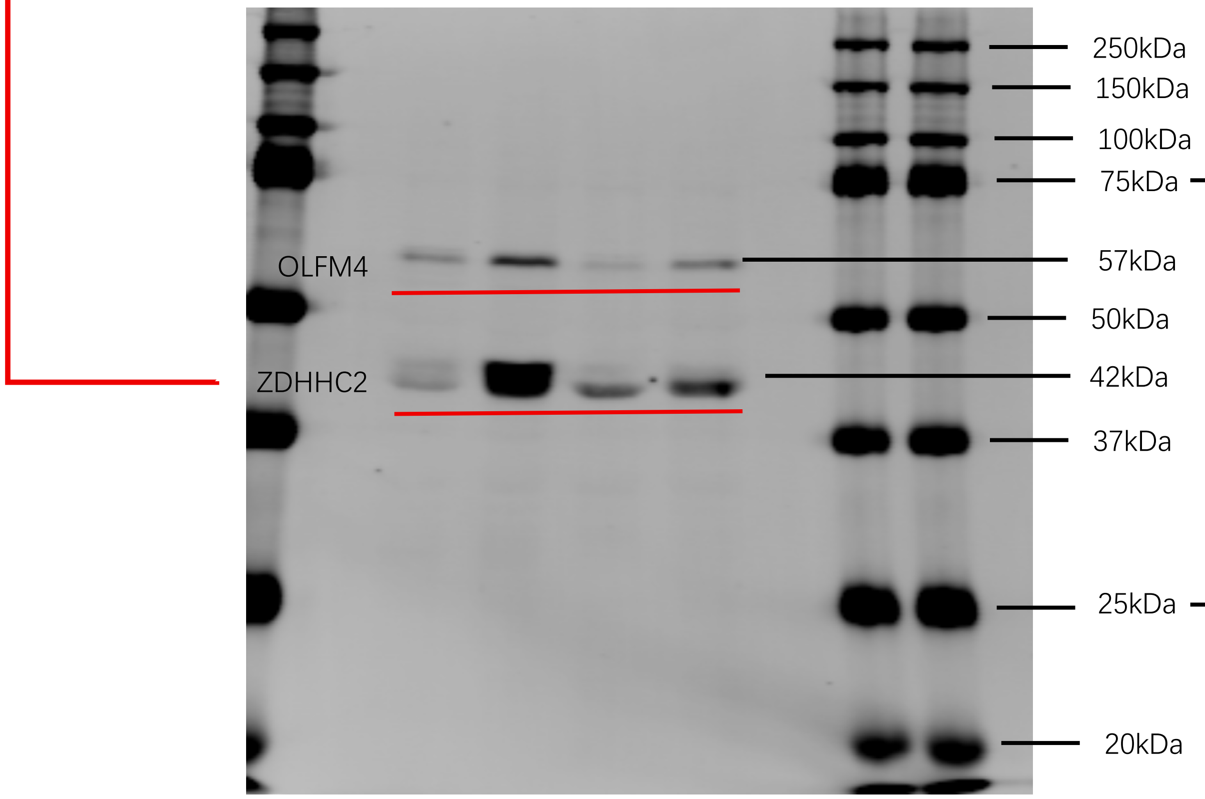

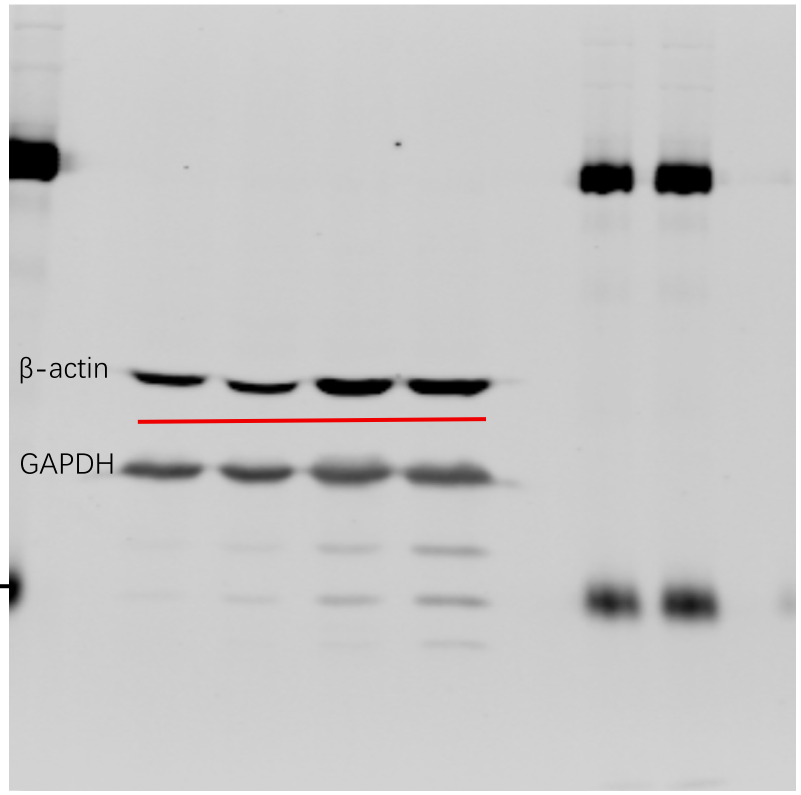

Precision Plus Protein™ Standards, #161-0374, BIO-RAD

Sample 2

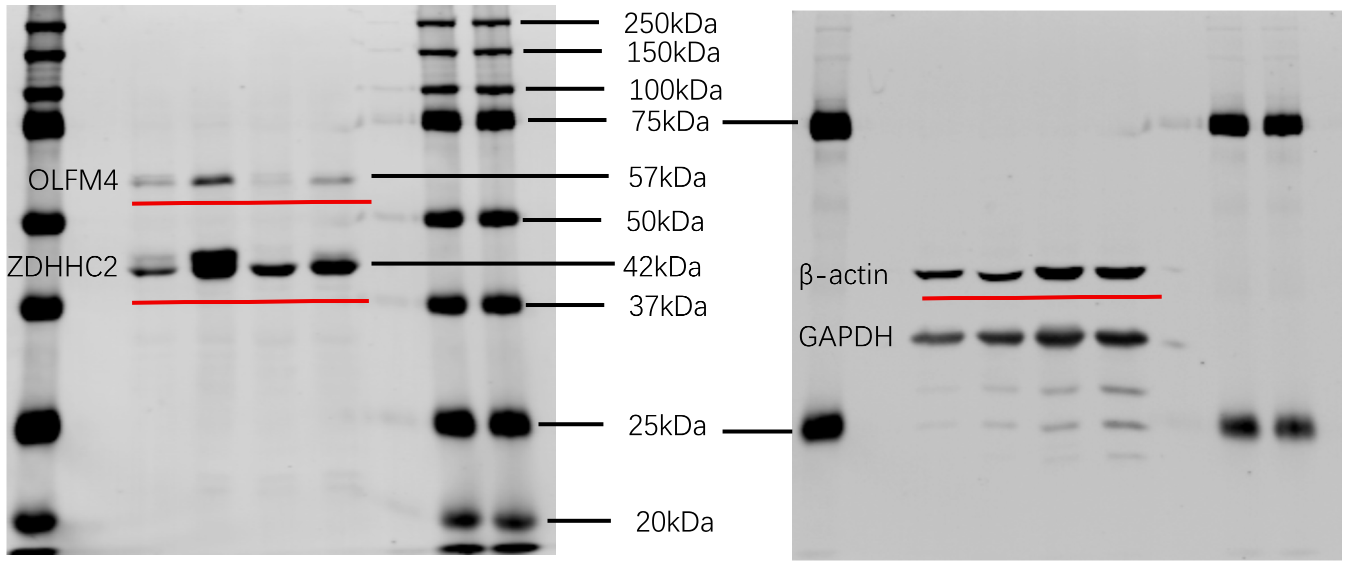

Precision Plus Protein^{TM} Standards, #161-0374, BIO-RAD

Sample 3

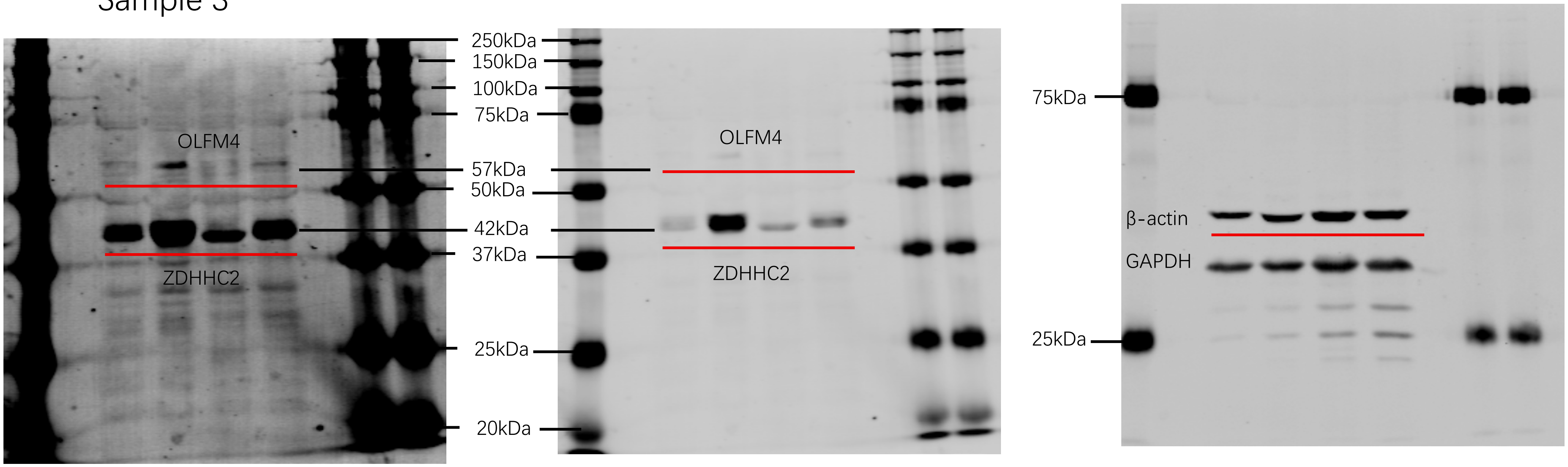

Supplement: Supplementary file 1 [file biomolecules-16-00880-s001.zip › RawWBimage_1.pdf]
