# Supplementary figures and images for: Single-Cell Transcriptomic Analysis Identifies an OLFM4-Associated Gastric Cancer Cell State with Palmitoylation-Related Signatures and Altered Metabolic Activities
Source: Biomolecules. 2026 Jun 15;16(6):880. doi: 10.3390/biom16060880 (PMC13296650; doi:10.3390/biom16060880)

Repeat 1

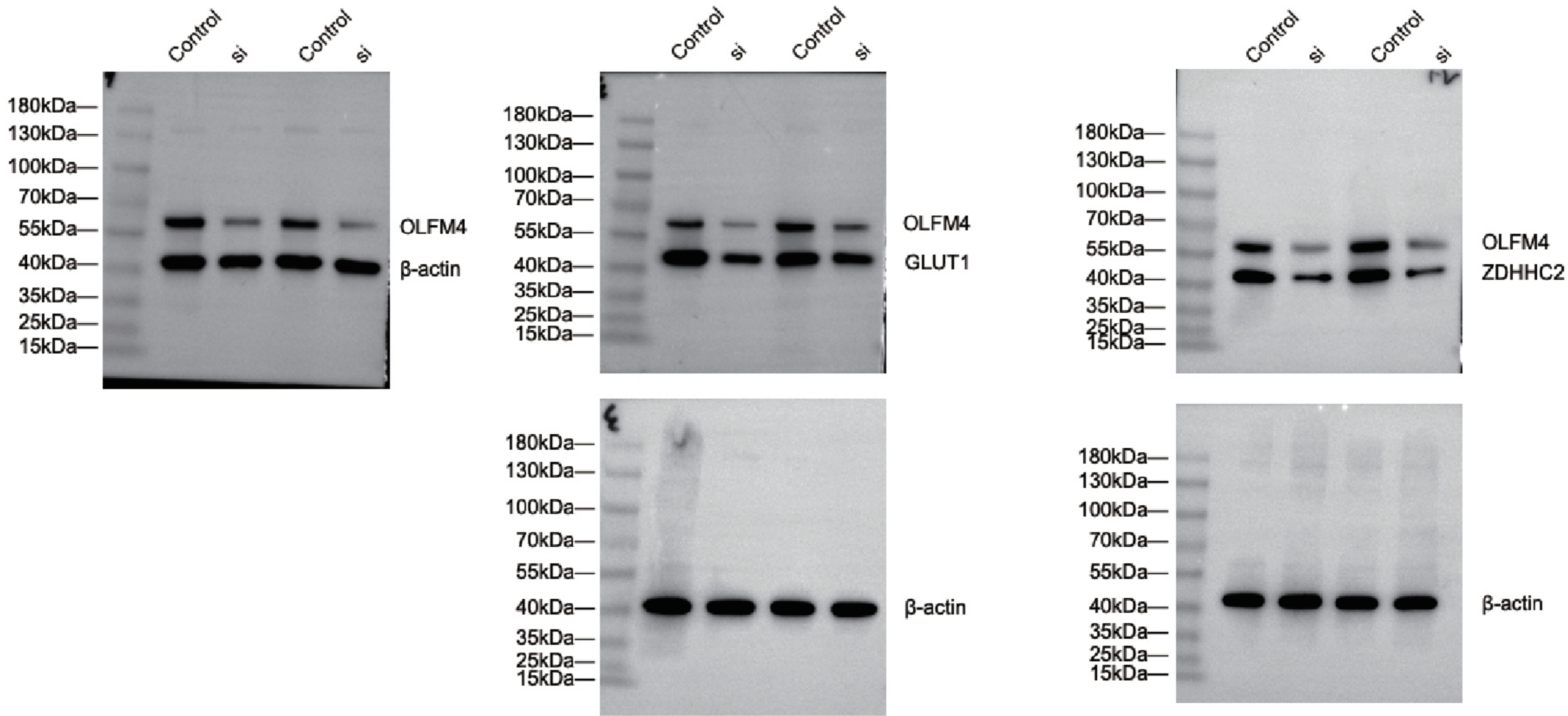

Repeat 2

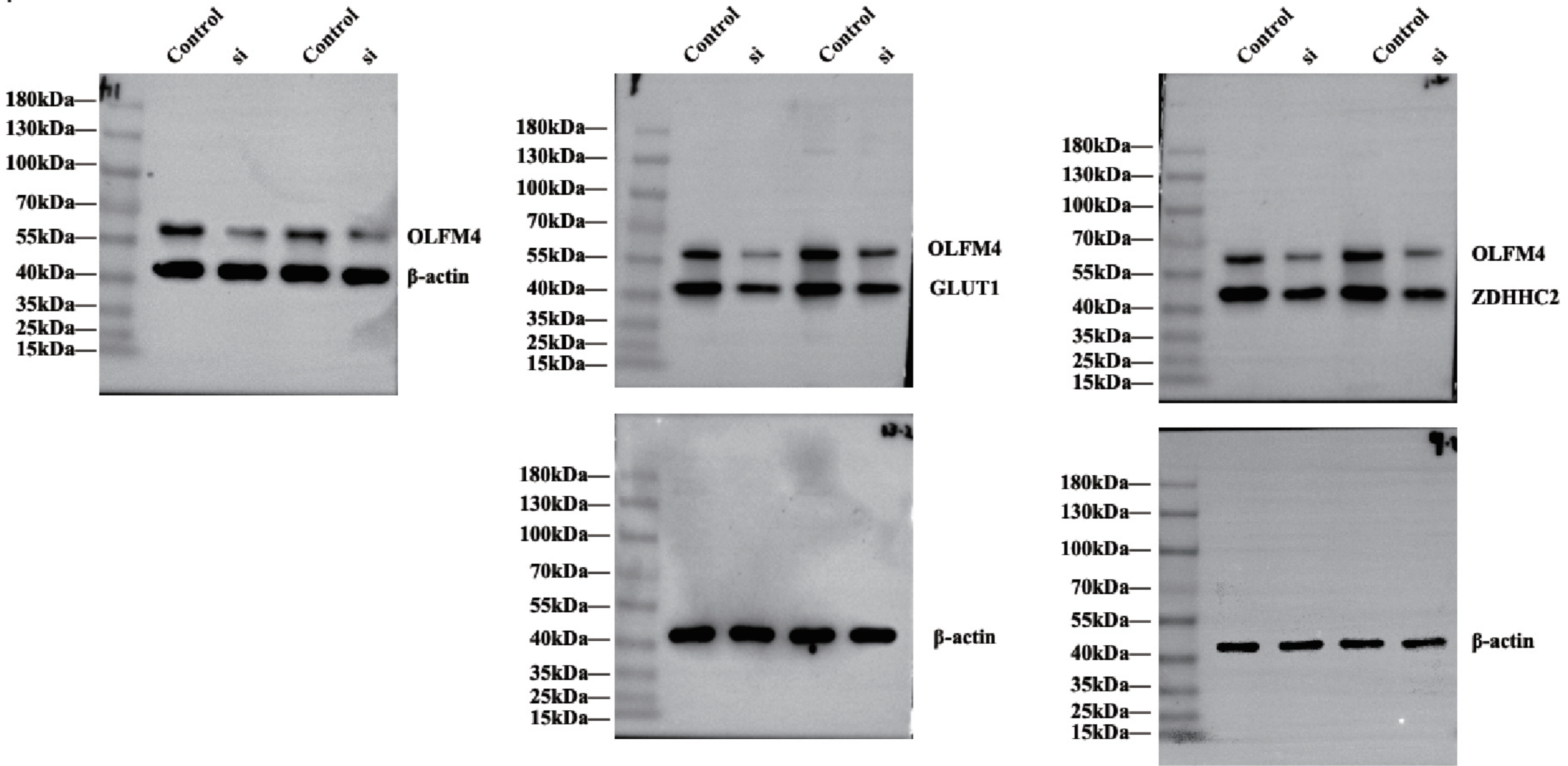

Repeat 3

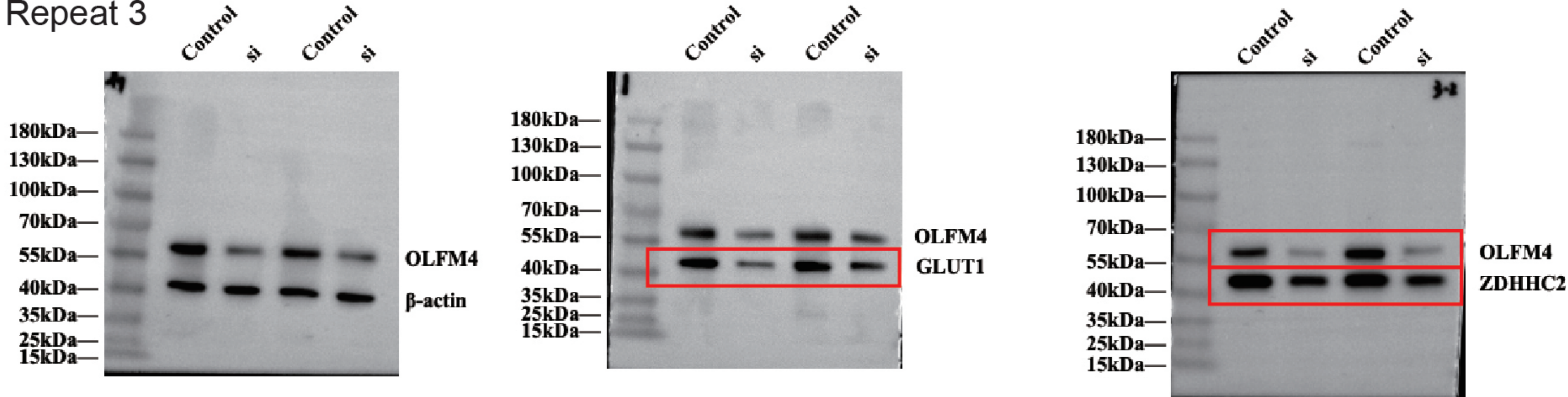

Figure 6H

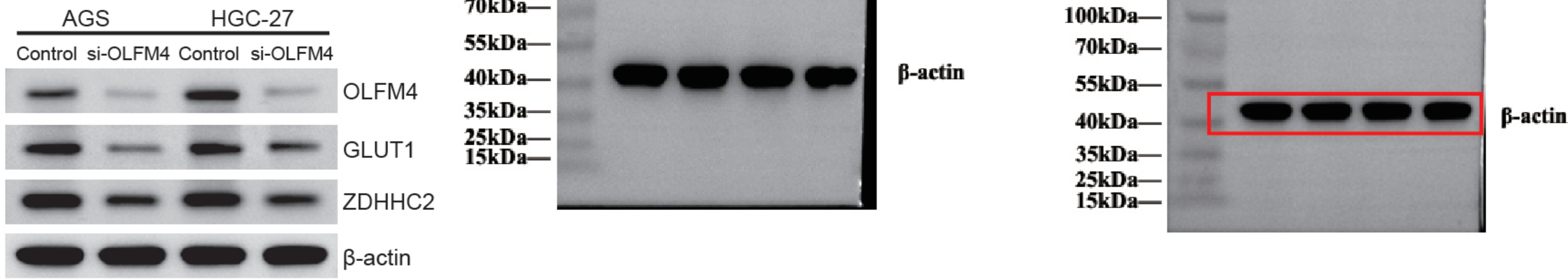

Supplement: Supplementary file 1 [file biomolecules-16-00880-s001.zip › si_RawWBimage_2.pdf]
